# A retrospective study on the clinical and pathological features of hepatic sarcomatoid carcinoma: Fourteen cases of a rare tumor

**DOI:** 10.1097/MD.0000000000030005

**Published:** 2022-08-05

**Authors:** Shuoshuo Ma, Chunshuang Li, Yang Ma, Xiaolei Wang, Dengyong Zhang, Zheng Lu

**Affiliations:** a Department of General Surgery, The First Affiliated Hospital of Bengbu Medical Collage; b Department of Pathology, The First Affiliated Hospital of Bengbu Medical College; c Department of Imaging, The First Affiliated Hospital of Bengbu Medical College, Bengbu, Anhui, China.

**Keywords:** diagnosis, hepatic sarcomatoid carcinoma, treatment

## Abstract

Hepatic sarcomatoid carcinoma is a rare liver malignancy with atypical clinical symptoms and a high degree of malignancy. To improve the understanding of this disease, we collected the clinical and pathological data of 14 patients with hepatic sarcomatoid carcinoma admitted to the First Affiliated Hospital and Second Affiliated Hospital of Bengbu Medical College from 2011 to 2021 and reviewed the relevant literature.

The clinical and pathological data of 14 patients with hepatic sarcomatoid carcinoma were collected from the electronic medical record system of the 2 hospitals. All clinical data were independently reviewed by 2 clinicians, and all pathological data were independently reviewed by 2 pathologists. At the same time, we reviewed the related literature on hepatic sarcomatoid carcinoma in Pubmed and CNKI.

This group of 14 patients, 10 males and 4 females, aged 50–77 years. The main symptoms of the patients were abdominal pain, bloating, anorexia, fatigue or weight loss in the upper abdomen, and 3 patients were asymptomatic. On imaging, hepatic sarcomatoid carcinoma manifests as heterogeneous mass with irregular shape and unclear boundary, and computed tomography (CT)/magnetic resonance imaging (MRI) enhanced scan shows progressive or persistent heterogeneous enhancement, marginal enhancement or annular enhancement, and central necrosis. The pathological features of hepatic sarcomatoid carcinoma are the proliferation of spindle cells and pleomorphic cells, which alternate with acinar cells.

Hepatic sarcomatoid carcinoma is more common in middle-aged and elderly patients, especially men, and has no characteristic clinical manifestations. Imaging examination and B-ultrasound-guided liver biopsy + immunohistochemistry can help diagnose. Radical surgery is the preferred method for hepatic sarcomatoid carcinoma, and postoperative adjuvant chemotherapy is expected to prolong patient survival.

## 1. Introduction

Hepatic sarcomatoid carcinoma (HSC) is a rare malignant tumor whose pathogenesis is still unclear. It is a hepatic malignancy composed of a combination of cancerous and sarcomatous components and accounts for <4% of all surgically resected hepatic malignancies.^[1]^ The age of onset of HSC is about 60 years old, and it is more common in men, and its incidence is 3–4 times higher than that in women.^[2,3]^ HSCs may be tumors of monoclonal origin, but their histological origin remains controversial. Compared with hepatocellular carcinoma and intrahepatic cholangiocarcinoma, HSCs are histologically poorly differentiated, have a high recurrence and metastasis rates, and thus have a poor prognosis.^[4]^ Due to the atypical clinical manifestations of HSC and the lack of characteristic tumor markers, imaging examination and B-ultrasound-guided liver biopsy + immunohistochemistry are important means of diagnosis. Surgery is currently the first choice for early HSC treatment, and adjuvant therapy includes radiotherapy, chemotherapy, hyperthermia, and interventional therapy. Now, the clinical and pathological characteristics of HSC are discussed based on the relevant data of the patients with hepatic sarcomatoid carcinoma diagnosed and treated in the First Affiliated Hospital and the Second Affiliated Hospital of Bengbu Medical College in the past ten years.

## 2. Materials and Methods

### 2.1. Diagnosis

Of the 14 patients in this group, 9 were confirmed by liver biopsy and 5 were confirmed by postoperative pathology. The punctured tissue or postoperative tissue samples were fixed in 10% formalin, dehydrated, embedded in paraffin, sectioned with 0.5 μm standard, stained with hematoxylin-eosin, sealed with neutral resin, and observed by electron microscope. The tumor tissue paraffin blocks were selected and sliced for immunohistochemistry. Vimentin (Vim), cytokeratin (CK), cytokeratin 7 (CK7), cytokeratin 19 (CK19), S-100, AE1/AE3, Ki-67 primary antibodies were purchased from the Cell Signaling Technology company. All diagnoses were independently assessed by 2 pathologists. In addition, we reviewed the relevant literature on hepatic sarcomatoid carcinoma in Pubmed and the Chinese National Knowledge Infrastructure (CNKI). Diagnostic criteria: combined with tumor tissue morphology and immunohistochemistry. (1) The morphological manifestations of tumor tissue were bidirectional expression of epithelial and mesenchymal tissues. (2) Immunohistochemistry showed positive epithelial component markers (CK, EMA, CEA, etc) and mesenchymal phenotypic markers (Vimentin, CD68, etc).

### 2.2. Treatment

Of the 14 patients, 9 were diagnosed with hepatic sarcomatoid carcinoma, 1 with hepatocellular carcinoma, 1 with intrahepatic cholangiocarcinoma, and 3 with liver malignancies before treatment. One patient was unable to tolerate major surgery because of poor cardiac function; so transcatheter arterial chemoembolization (TACE) was performed 3 times before and after. The drugs used were tegafur 1 g + THP30 mg + lipiodol 8 ml, tegafur 1 g + THP30 mg + lipiodol 8 ml, tegafur 1 g + oxaliplatin 150 mg + THP30 mg + lipiodol 6 ml. One patient lost the opportunity for surgery because of multiple metastases in the pancreas, lung, and neck, and only symptomatic and supportive treatment was given. One case was treated with pirarubicin + cisplatin for 1 course of chemotherapy because of multiple retroperitoneal metastases, but the curative effect was poor. In 1 case, only symptomatic and supportive treatment was selected due to personal and family factors. One case of poor nutritional status, only symptomatic treatment. One case of intrahepatic metastasis complicated with peritoneal metastasis was selected for 2 cycles of chemotherapy (epirubicin + cisplatin). Six patients underwent radical surgery, 1 patient underwent right posterior lobectomy, 1 patient underwent right hepatectomy, 1 patient underwent segment IV tumor resection, and 1 patient underwent hepatic lobectomy (middle hepatectomy) + Cholecystectomy + hepatoduodenal ligament lymph node dissection, 1 patient underwent left lateral hepatic lobectomy + partial gastrectomy + abdominal adhesion release, 1 patient underwent liver segment VI/VII/partial VIII resection + Diaphragmatic metastases resection + diaphragm repair + cholecystectomy. In 4 cases, a reverse “L”-shaped incision was used in the upper abdomen, and 2 cases were made with an oblique incision under the right upper abdominal costal margin. Irregular resection was performed in 1 case and regular resection in 5 cases. The average blocking time of the first porta hepatis was 38.4 minutes (range: 10–90 minutes). The average intraoperative blood loss was 417 mL (range: 100 mL–800 mL), and 4 cases of intraoperative blood transfusion, including 1 case of whole blood, 2 cases of red blood cells, and 1 case of depleted leukocytes, the average plasma input volume was 445 mL (range: 400 mL–480 mL). The mean operative time was 330 minutes (range: 175–679 minutes), and the mean postoperative hospital stay was 10.8 days (range: 8–15 days).

### 2.3. Statistical analysis

Categorical variables were represented as the number of cases (rate) and analyzed using a Chi-square test or the Fisher exact test, while continuous variables were represented as medians with an interquartile range (IQR) and compared using Mann–Whitney U tests. Statistical significance was defined as 2-sided *P* values < .05, and all statistical analyses were performed using the Statistical Package for the Social Sciences version 25.0 (SPSS 25.0, Chicago, IL).

## 3. Results

### 3.1. Clinical features

There were 14 patients in this group, including 10 males and 4 females, with an average age of 61.2 years (range: 50–77 years). The tumor was located in the left lobe of the liver in 4 cases and the right lobe in 8 cases. Eight cases were single, 4 cases were intrahepatic multiple metastases, and 5 cases were extrahepatic metastasis, which was respectively transferred to retroperitoneal lymph nodes, greater omentum lymph nodes and mesenteric lymph nodes, diaphragm, right lung and rectus abdominis, both lungs, peripancreatic and neck. The detailed clinical characteristics of the patients are shown in Table 1.

**Table 1:** Clinical outcomes of 14 patients with hepatic sarcomatoid carcinoma.

Cases	Age	Gender	Symptoms	Tumor location	Maximum diameter of tumor (cm)	Carcinomatous component	Treatment	OS (mo)
Case 1	74	Female	None	Right lobe	8.0	CHOL (small duct type)	TACE	8
Case 2	65	Female	Abdominal pain, Bloating, Anorexia,Weight loss	Right lobe	4.0	CHOL (small duct type)	Surgery	7
Case 3	50	Male	Anorexia, Fever, Weight loss	Right lobe	9.5	CHOL (small duct type)	Surgery + TAI	5
Case 4	50	Male	Weight loss	Right lobe	5.2	CHOL (small duct type)	Supportive care	3
Case 5	64	Male	Abdominal pain, bloating, anorexia	Left lobe	8.5	CHOL (small duct type)	Chemotherapy	4
Case 6	50	Male	None	Right lobe	2.2	CHOL (small duct type)	Surgery + TACE + chemotherapy + radiotherapy	35
Case 7	64	Female	Abdominal pain, bloating, weight loss	Left lobe	—	CHOL (small duct type)	Supportive care	5
Case 8	53	Male	—	—	—	—	—	—
Case 9	77	Female	Abdominal pain, Anorexia	Left lobe	—	CHOL (small duct type)	Supportive care	0
Case 10	54	Male	Bloating, Nausea	Right lobe	3.2	CHOL (small duct type)	Chemotherapy	5
Case 11	63	Male	—	—	—	—	—	—
Case 12*	65	Male	Fatigue, anorexia, jaundice	Right lobe	4.2	CHOL (large duct type)	Surgery + Chemotherapy	11
Case 13*	63	Male	Fever	Left lobe	11.2	CHOL (small duct type)	Surgery	6
Case 14*	65	Male	None	Right lobe	10.6	CHOL (small duct type)	Surgery	3

### 3.2. Comorbidities

Some patients had comorbid symptoms, including fatty liver (1/14, 7%), pleural effusion (1/14, 7%), gallbladder stones (2/14, 14%), chronic nonatrophic gastritis (2/14), 14%), chronic superficial mucosal gastritis (1/14, 7%), history of hypertension (4/14, 29%), history of diabetes (3/14, 21%), history of stroke (2/14, 21%) 14%), kidney stones (1/14, 7%), renal cyst (2/14, 14%), pancreatic cyst (1/14, 7%), spleen cyst (1/14, 7%), intergastric Plasma tumor (1/14, %), history of LC surgery (2/14, 14%) and gynecological surgery (1/14, 7%). 3 patients (21%) were asymptomatic, and 2 patients (14%) had unknown medical history.

### 3.3. Clinical manifestations

There were no characteristic clinical manifestations in patients with hepatic sarcomatoid carcinoma, including abdominal pain and distension in 4 cases (29%), anorexia in 3 cases (21%), fever in 2 cases (14%), nausea in 2 cases (14%), and jaundice in 1 case (7%), fatigue in 1 case (7%), weight loss in 5 cases (36%), and asymptomatic in 3 cases (21%).

### 3.4. Imaging performance

**Figure 1.**
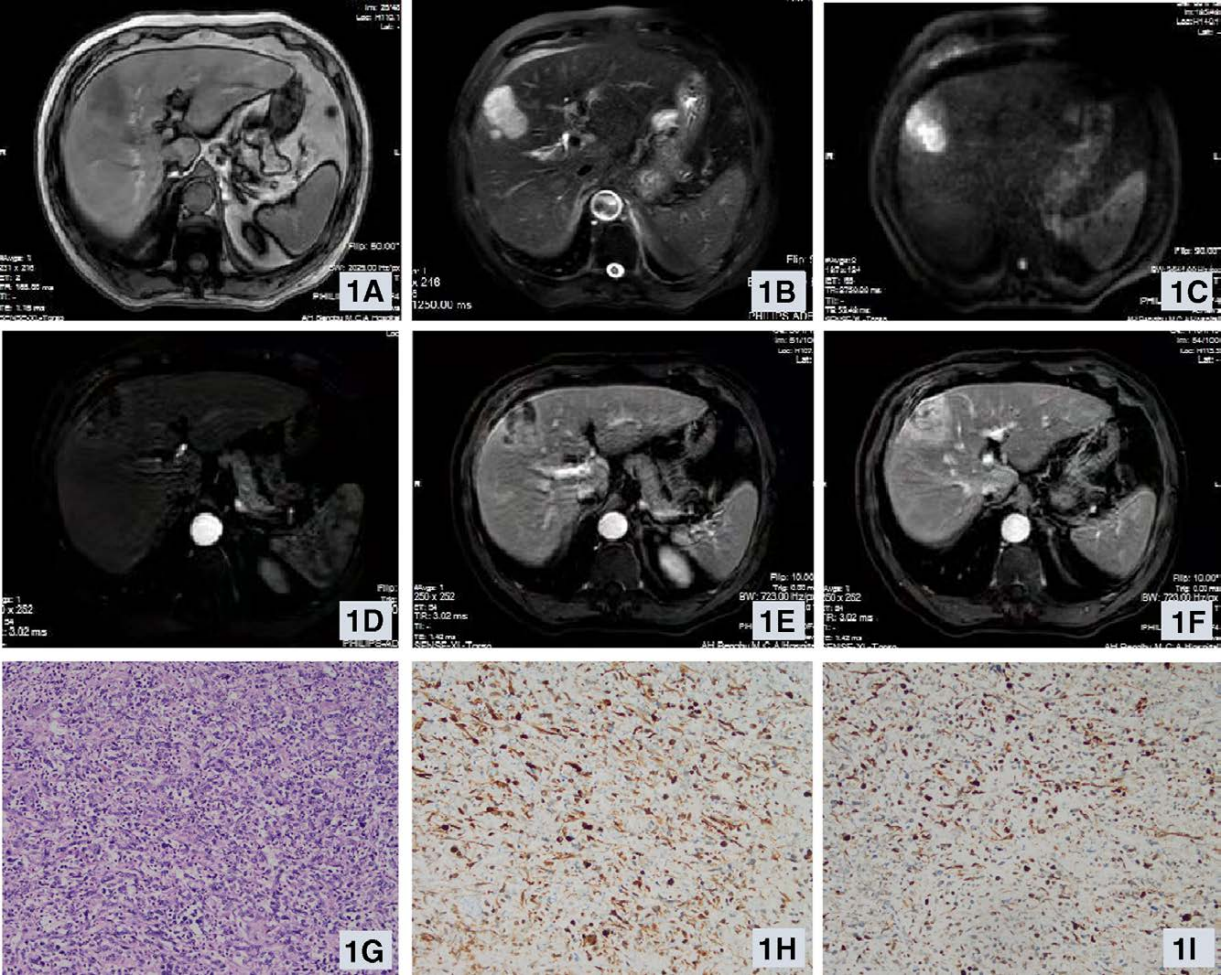
Relevant clinical data of a patient with hepatic sarcomatoid carcinoma (Case 12): preoperative enhanced MRI images showed a mass of mixed T1 signal (1A) and slightly longer T2 signal (1B) under the capsule of the right anterior lobe of the liver, with a size of about 3.3 cm*3.4 cm*4.2 cm, partially protruding from the liver capsule, showing obvious uneven high signal on DWI (1C). On contrast-enhanced scan, arterial phase enhancement (1D), portal venous phase (1E) and venous phase (1F) washout, and pseudocapsules can be seen. Postoperative pathological examination images (HE staining × 200) (1G), immunohistochemical examination images, epithelial keratin positive (immunohistochemical staining × 200) (1H), vimentin positive (immunohistochemical staining × 200) (1I).

**Figure 2.**
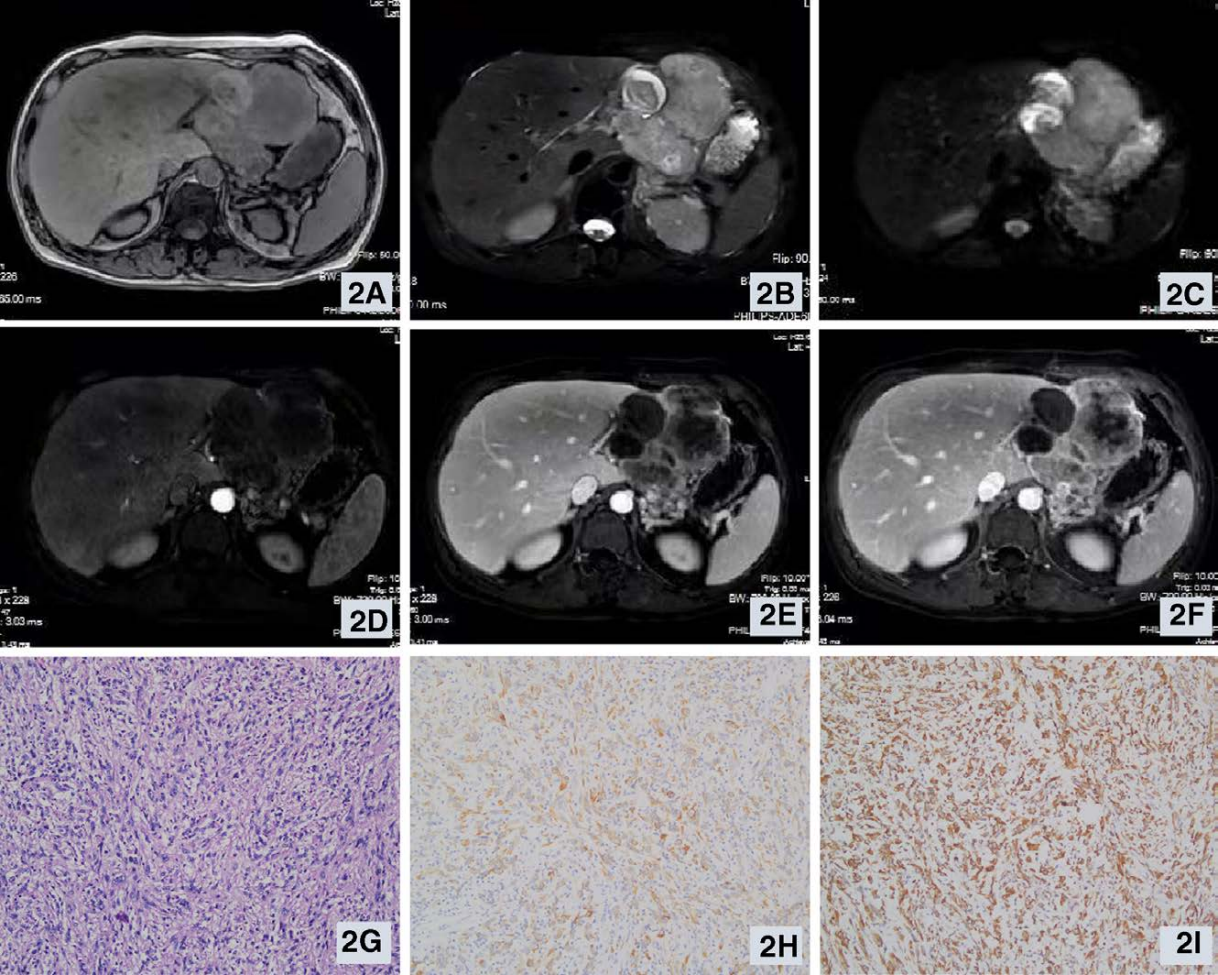
Relevant clinical data of a patient with hepatic sarcomatoid carcinoma (Case 13): preoperative enhanced MRI image, abnormal signal of long T1 (2A) and long T2 (2B) in the liver and stomach space, the size is about 9.5 cm*8.3 cm, a round-like short T1 and long T2 signal can be seen in it, the boundary is clear, the boundary between the lesion and the left hepatic lobe is unclear, DWI shows high signal (2C), and the enhancement shows progressive and heterogeneous enhancement (2D, 2E, and 2F); postpathological examination images, microscopic images (HE staining × 200) (2G); immunohistochemical examination images, epithelial keratin positive (immunohistochemical staining × 200) (2H), vimentin positive (immunohistochemical staining × 200) (2I).

Of 14 patients, 11 patients underwent CT plain scan + enhanced before treatment, and hepatic sarcomatoid carcinoma showed a mass, round or sheet-like low-density shadow on CT imaging. Five cases had blurred borders and 1 case had clear borders. 4 cases showed uneven density, and 1 case showed gas-liquid level with multiple segmentations. Enhanced CT scan showed persistent or progressive heterogeneous enhancement in 7 cases, and transient enhancement in 1 case. In 5 cases, the intrahepatic and extrahepatic bile ducts were not dilated, and the intrahepatic bile ducts were slightly dilated in 1 case. There were no enlarged lymph nodes in the retroperitoneum in 4 cases, and multiple lymph nodes in the retroperitoneum were found in 1 case. MRI examination was performed in 4 cases, and the lesions were irregular masses with unclear boundaries. 1 case showed mixed T1 signal (Fig. 1A), slightly long T2 signal (Fig. 1B), 3 cases showed long T1WI (Fig. 2A) and long T2WI signal(Fig. 2B), and DWI showed a high signal(Fig. 1C and Fig. 2C). Enhanced MRI scans showed heterogeneous enhancement (Fig. 1D–F and Fig. 2D–F) in 2 cases and annular enhancement in 2 cases. Pseudocapsule formation occurred in 1 case, intratumoral hemorrhage in 1 case, and intratumoral necrosis in 2 cases. Four cases of intrahepatic and extrahepatic bile ducts were not dilated, 3 cases of retroperitoneal lymph nodes showed no obvious enlargement, 1 case of retroperitoneal and mesenteric root lymph node enlargement. One patient underwent PET/CT examination, and the results showed that the foci of increased fluorodeoxyglucose metabolism were suggestive of malignancy. The imaging examination results of 11 cases showed a liver mass, including 4 cases of hepatocellular carcinoma, 1 case of intrahepatic cholangiocarcinoma, 1 case of intrahepatic metastasis, 2 cases of liver abscess, 1 case of hepatic hemangioma, and 2 cases of hepatic hemangioma. Hepatic sarcomatoid carcinoma was not considered in all cases.

### 3.5. Laboratory tests

Carcinoembryonic antigen and alpha-fetoprotein were detected in 11 cases, carbohydrate antigen 15–3 in 10 cases, and prostate-specific antigen in 7 cases, all within the normal range. Carbohydrate antigen 19-9 was detected in 11 cases and carbohydrate antigen 125 was detected in 4 cases, each of which exceeded the normal range (52.18IU/mL and 100.7IU/mL, respectively). Five cases of hepatitis B surface antigen immunological indicators were positive, and 1 case of alanine aminotransferase combined aspartate transferase was higher than the normal value (82U/L and 124U/L respectively). Alkaline phosphatase was increased in 8 cases (127U/L–290U/L). Total bilirubin was elevated in 2 cases (24.7 μmol/L and 22.6 μmol/L, respectively), and direct bilirubin was elevated in 5 cases (9.6 μmol/L–14.9 μmol/L). The albumin concentration was decreased in 9 cases (28.9 g/L–39.9 g/L), and the white ball ratio was decreased in 8 cases (0.6–1.12). Nine cases of hypersensitivity protein increased to varying degrees (6.70–298.06) ng/L, and the total cholesterol concentration of 1 case was slightly increased (5.31 mmol/L). Detailed laboratory results can be found in Table 2.

**Table 2:** Biomarkers of 14 patients with hepatic sarcomatoid carcinoma.

Cases	CEA	AFP	CA15-3	CA19-9	ALT	AST	ALP	TBIL	ALB	TC	CRP	HBsAg
	(ng/mL)	(ng/mL)	(IU/mL)	(IU/mL)	(U/L)	(U/L)	(U/L)	(μmol/l)	(g/L)	(mmol/L)	(mg/L)	
Case 1	2.37	< 15	25.20	32.95	18.00	17.00	52.00	9.70	39.90	4.29	2.79	(−)
Case 2	1.47	< 15	7.50	52.18	12.00	27.00	115.00	7.40	38.80	5.31	18.37	(+)
Case 3	1.15	1.88	5.55	8.81	82.00	124.00	264.00	20.70	28.90	3.00	164.00	(−)
Case 4	1.68	2.50	6.59	9.47	27.00	40.00	290.00	24.70	41.40	3.45	150.90	(−)
Case 5	2.13	2.43	17.60	25.70	19.00	31.00	127.00	12.20	48.30	4.42	49.58 (+)	
Case 6	2.53	6.47	5.30	2.13	28.00	18.00	63.00	14.30	39.70	3.68	0.22	(+)
Case 7	1.86	2.13	13.30	18.40	40.00	39.00	93.00	10.00	34.70	3.25	6.70 (+)	
Case 8	—	—	—	—	—	—	—	—	—	—	—	—
Case 9	—	—	—	—	10.00	23.00	160.00	18.80	30.30	4.99	298.06	(−)
Case 10	<0.5	0.98	18.20	<2.00	25.00	32.00	206.00	5.20	30.10	3.99	190.20	(−)
Case 11	—	—	—	—	—	—	—	—	—	—	—	—
Case 12*	1.19	1.54	15.22	5.97	28.00	24.00	184.00	16.70	33.50	3.65	154.60	(+)
Case 13*	1.24	4.47	18.10	6.32	37.00	18.00	188.00	6.50	32.10	2.64	215.70	(−)
Case 14*	1.35	4.04	—	0.86	23.00	25.00	158.00	22.60	45.80	3.70	—	(−)

### 3.6. Ultrasound-guided liver biopsy

12 patients underwent liver biopsy under the guidance of B-ultrasound, and the specimens were histopathologically examined by HE staining, 11 patients were suggested to be a liver malignancy, 1 patient was suggested to be a liver abscess, and repuncture suggested liver malignancy. Immunohistochemical examination of 10 cases showed that the tumor tissue had both epithelial tumor components and spindle cell sarcoma components. Nine cases expressed Vim, 6 cases expressed cytokeratin CK, 4 cases expressed CK7, and 4 cases expressed CK19. In addition, 1 case expressed CD34, 1 case expressed EMA, and 1 case expressed AE1/AE3, Des and β-catenin. Seven cases were positive for Ki-67, and the value-added index was about 25 to 70%. Pathological examination results showed that 9 cases were hepatic sarcomatoid carcinoma, 1 case was intrahepatic cholangiocarcinoma carcinoma, 1 case was hepatocellular carcinoma, and 1 case only suggested liver malignancy.

### 3.7. Postoperative pathology

In 6 cases of postoperative pathology, the average maximum diameter of surgically resected tumor specimens was about 7.1 cm (range: 2.5–11.0 cm), and the resection margins were all negative. The surface of the tumor specimen was gray-white with the naked eye, the texture was tough, and necrosis was seen on the cut surface. The pathological features of hepatic sarcomatoid carcinoma are mainly the proliferation of spindle cells and pleomorphic cells, which alternate with acinar cells (Fig. 1G and Fig. 2G).^[5]^ Combined with immunohistochemistry, hepatic sarcomatoid carcinoma was diagnosed. 4 cases were positive for cytokeratin (Fig. 1H and Fig. 2H), 5 cases were positive for vimentin (Fig. 1I and Fig. 2I), 2 cases were positive for CK7, 3 cases were positive for CK19, 1 case was positive for S-100, and 2 cases were positive for AE1/AE3. Four cases expressed Ki-67, and the cell proliferation index was about 55–70%. Pathological examination results showed that they were all hepatic sarcomatoid carcinomas. Detailed immunohistochemical indicators can be seen in Table 3.

**Table 3:** Immunohistochemical molecular markers of 14 patients with hepatic sarcomatoid carcinoma.

Cases	Positive	Negative
Case 1	Vim (3+)/CK7 (2+)	CK20 (-)/AFP(-)/CD34(-)/HMB-45(-)/SMA(-)/hepatocyte(-)
Case 2	CK7(2+)/CK19(+)/Vim(+)/S100(+)	CD34(-)/CD117(-)/Dog-1(-)/SMA(-)
Case 3	CK(+)	AFP(-)/hepatocyte(-)/S-100(-)/SMA(-)/HMB45(-)/CD117(-)/Dog-1(-)
Case 4	CK(2+)/Vim(2+)	CD117(-)/Dog-1(-)/SMA(-)/HMB-45(-)/CD34(-)/Melan-A(-)/S-100(-)/
		Des(-)/hepatocyte(-)/CEA(-)/CK19(-)/Gpc(-)
Case 5	CK(+)/Vim(+)	CK7(-)/CK19(-)/GDC(-)/hepatocyte(-)
Case 6	CK19(3+)/CK(+)/Vim(+)/GPC-3(-/+)	CD34(-)/hepatocyte(-)/CK7(-)/AFP(-)
Case 7	CK(2+)/Vim(2+)/CK19(+)	CD31(-)/CD34(-)/CEA(-)/Villin(-)/hepatocyte(-)
Case 8	CK(-/+)/Vim(2+)/CK19(-/+)	CD34(-)/CD31(-)/CD117(-)/hepatocyte(-)
Case 9	CK(2+)/CK7(+)/CK19(+)/Vim(2+)	TTF-1(-)/Napasina(-)
Case 10	CK(+)/Vim(+)	GPC(-)/hepatocyte(-)/SMA(-)/CK19(-)/CD163(-)
Case 11	CK(2+)/CK7(2+)/EMA(2+)	HMB(-)/MelenA(-)/SMA(-)/CD117(-)/S-100(-)/MDM2(-)/CD34(-)
		β-catenin(-)/CK20(-)/CK19(-)/P63(-)/hepatocyte(-)
Case 12*	CK(+)/CK19(-/+)/Vim(2+)	SOX10(-)/CEA(-)/Heppar(-)/hepatocyte(-)/LCA(-)
Case 13*	AE1/AE3(3+)/CDK4(+)Vim(+)	CK(-)/CD117(-)/Dog(-)/CD34(-)/SMA(-)/GPC(-)/Heppar(-)
Case 14*	AE1/AE3(+)/CK7(+)/Vim(+)/CK19(+)	GPC(-)/Hep-1(-)/SMA(-)/β-catenin(-)

### 3.8. Prognosis

The follow-up of this group ended in November 2021, and the follow-up time of the whole group was 1–35 months. Eleven patients died, of which 2 were due to pulmonary infection, and the remaining 9 were tumor recurrence and metastasis. The overall survival time of patients with tumor resection was (10.8 ± 12.3) months (range: 1–35 months), the overall survival time of patients with chemotherapy alone was 4 months and 5 months, respectively, and the overall survival time of patients with symptomatic supportive care was (2.7 ± 2.5) months (range: 0–5 months).

## 4. Discussion

Sarcomatoid carcinoma (SC) is relatively rare in clinical practice and can occur in multiple organs throughout the body,^[6–9]^ but the lung and bladder are the most common sites.^[10,11]^ HSC is an uncommon malignancy, accounting for 1.8% of all surgically resected liver malignancies and 3.9–9.4% of autopsy cases.^[12,13]^ It is a mixed tumor composed of cancerous and sarcomatoid components. According to the different cancer components, it is mainly divided into sarcomatoid hepatocellular carcinoma (SHC) and intrahepatic sarcomatoid cholangiocarcinoma (ISCC) and undifferentiated carcinoma.^[14,15]^ The sarcoma component consists of poorly differentiated cells that grow rapidly with new blood vessels, and the feeding vessels cannot adequately supply the rapidly growing malignant cells, resulting in necrosis within the tumor. HSCs are highly aggressive, and the presence of SCs is thought to be closely associated with more aggressive tumor biology, more frequent metastasis, lower resectability, and frequent postoperative recurrence.^[4,16,17]^ HSC mostly occurs in the right lobe of the liver, and rarely occurs in the left lobe of the liver^[1]^; the age of onset is mostly around 60 years old, and it is common in men, with an incidence of 3 to 4 times that of women.^[2,3]^ There were 14 patients in this group. The age of onset (61.21 ± 8.66) years, gender (male: female = 10:4), and site of occurrence (right: left = 8:4) were basically consistent with the previous reports.

HSC lacks typical clinical manifestations in the early stage. Abdominal pain is the most common symptom of HSC, followed by fatigue, fever, and jaundice.^[18]^ Some patients may also show gastrointestinal symptoms such as abdominal distension, nausea, vomiting, and anorexia.^[19]^ The above symptoms were manifested in different proportions in this group of patients, among which weight loss was the most common symptom, followed by abdominal pain and bloating. HSC has a high pathological grade,^[2]^ poor tumor differentiation,^[2,3,20]^ is prone to local invasion and distant metastasis, and has a poor prognosis. Zhang et al^[1]^ found in a study that up to 47.1% of patients developed intrahepatic metastasis and 93% of patients developed extrahepatic metastasis. The most common metastatic organs were lung, peritoneum, pleura, pancreas, adrenal gland, intestine and Spleen.^[21]^ In this group of patients, there were 4 cases of intrahepatic multiple metastases and 6 extrahepatic metastasis. Among them, there were 2 cases of lung metastasis (1 case with peripancreatic lymph node and cervical lymph node metastasis, 1 case with rectus abdominis metastasis), 2 cases with peritoneal metastasis, 1 case with retroperitoneal lymph node enlargement and fusion into a mass, and 1 case with diaphragmatic muscle metastasis. Zhang et al^[1]^ also pointed out that about half of HSC patients have HBV infection, and HBV infection helps to differentiate HSC from HCC, especially in HBV-negative cases, because 92.0% of HCC patients have HBV infection. In this group of patients, 5 patients tested positive for hepatitis B surface antigen. Several studies have also pointed to a potential relationship between chronic liver disease and HSC.^[4,15,22]^ In our study, we also found that the liver function of the patients was damaged to varying degrees, manifested as prolonged PT (9 cases), elevated alkaline phosphatase (8 cases), inversion of white ball ratio (8 cases), alanine aminotransferase and increased aspartate aminotransferase (1 case). In addition, most patients had abnormally increased C-reactive protein (CRP), which may be related to the high degree of malignancy and strong invasive ability of HSC. Tumor markers such as CEA, AFP, CA15-3, and CA19-9 have very limited diagnostic value for HSC, and are positive indicators in a small number of patients. Studies have shown that the incidence of elevated CA19-9 and CEA in patients with hepatic sarcomatoid carcinoma is significantly higher than that in HCC patients, while the incidence of elevated AFP is much lower than that in HCC,^[1]^ which is helpful for the differential diagnosis of HSC and HCC.

Imaging examination plays an important role in the diagnosis of HSC, but it is easily confused with benign and malignant diseases such as atypical liver abscess, hepatocellular carcinoma, and intrahepatic cholangiocarcinoma. In this group of patients, 2 cases were misdiagnosed as liver abscess, 1 case of hepatocellular carcinoma, and 1 case of intrahepatic cholangiocarcinoma. In an imaging study including 17 HSCs, 50 HCCs, and 50 ICCs, the investigators pointed out that HSCs were longer than HCCs, were more prone to T2-weighted heterogeneity, and showed more persistent or persistent enhancement patterns than HCCs. Progressive enhancement, while HCC is often manifested as “fast in and fast out” of contrast media. In addition, HSCs are more prone to dilation of adjacent bile ducts, intrahepatic metastasis and lymphadenopathy. Compared with ICC, target signs and pseudo capsules are less common in HSC, and intratumoral hemorrhage is more common. In conclusion, HSC should be highly vigilant when the liver mass has the following characteristics: (1) The tumor index AFP is in the normal range, the density/signal in the CT/MRI scan is uneven, the enhancement is progressive or persistent, and the central necrosis is (2) with adjacent bile duct dilatation, multiple intrahepatic metastases, lymphadenopathy, vascular invasion or intratumoral hemorrhage; (3) lobulated, marginal arterial phase enhancement, peripheral washout, central delayed enhancement. For cases suspected to be hepatic sarcomatoid carcinoma, it is recommended to perform a biopsy of the liver under the guidance of B-ultrasound, plus immunohistochemistry. Microscopically, HSCs consist of spindle cells and ultrastructurally, immunohistochemically, and morphologically identifiable epithelial components.^[23]^ Immunohistochemical staining showed that the sarcomatoid component marker Vim was positive, and the epithelial component markers CK, epithelial membrane antigen (EMA), AE1/AE3, etc were positive.^[24,25]^ However, we should be alert to the possibility of misdiagnosis due to small and unrepresentative puncture samples.

Surgery is the preferred method for the treatment of HSC,^[19]^ and radical surgery can significantly prolong the overall survival of patients with HSC.^[26,27]^ In this group, 6 patients who received radical surgery had an overall survival time of (10.8 ± 12.3) months, which was longer than that of patients who received chemotherapy alone or supportive care (3 ± 1.87 months). Regular liver resection was performed in 5 cases, with an average tumor-free survival period of (12.33 ± 7.09) months; 1 case with irregular resection had a tumor-free survival period of 2 months. In this group of 6 patients undergoing surgery, 1 patient underwent lymph node dissection. The tumor-free survival and overall survival were both 11 months, and 5 patients did not undergo lymph node dissection. The tumor-free survival was (9 ± 9.5) months. Overall survival period (16 ± 16.46) months. Given the small sample size and patients receiving different treatment methods such as chemotherapy and interventional therapy after surgery, the effect of regular liver resection or lymph node dissection on the survival of patients cannot be concluded, which requires more clinical research. It is worth mentioning that among the 6 patients in this group who underwent radical resection, 3 patients received postoperative adjuvant chemotherapy, the disease-free survival time was (11.0 ± 9.0) months, and the overall survival time was (17.0 ± 15.9) months; Of the 3 patients who underwent surgery only, 2 recurred after 3 and 4 months, respectively, and the overall survival time was (5.3 ± 2.1) months. The differentiation of HSC is poor, and half of the patients have already developed intrahepatic metastasis and multiple extrahepatic metastases at the time of consultation, and lost the opportunity for surgery. Some studies have pointed out that systemic chemotherapy and local radiotherapy can be selected for patients whose tumors cannot be surgically removed.^[28]^ Due to the rarity of this tumor type and the lack of large studies, the exact effects of radiotherapy and chemotherapy are unknown. In this group of cases, 2 patients underwent chemotherapy alone, and the chemotherapy regimens were both pirarubicin + cisplatin. The overall survival time of the patients was 4 months and 5 months, respectively. This was not significantly different from the mean overall survival (4 months) of patients treated with symptomatic and supportive care. Studies have reported that HSCs are hypovascular tumors,^[29]^ and TACE is a risk factor associated with poor prognosis.^[30]^ For patients with unresectable intrahepatic recurrence, TACE can help prolong the survival of patients,^[31]^ which may be due to the rich blood supply of new tumors and the strong dependence of tumors on blood vessels. In this group, 2 patients underwent TACE, and 1 patient underwent TACE intervention for a total of 3 times before and after (the overall survival time was 8 months). The other 1 patient, who received 2 adjuvant TACE after surgery (disease-free survival of 20 months and overall survival of 35 months), had longer survival than other patients with chemotherapy alone or supportive care (3 ± 1.87 months), which may be related to the individual differences. In addition, some studies have pointed out that camrelizumab,^[32]^ apatinib,^[33]^ etc in the treatment of pulmonary sarcomatoid can alleviate the disease progression, but the application of immunotherapy in hepatic sarcomatoid carcinoma has not been reported.

## 5. Strengths and limitations of this study

1. This article comprehensively analyzes the clinical manifestations, imaging manifestations, pathological features, diagnosis, treatment and follow-up of hepatic sarcomatoid carcinoma;
2. This group of patients involves a variety of treatment methods, which can provide a reference for clinical practice;
3. The number of cases is too small, which has certain limitations.

## 6. Conclusions

To sum up, the diagnosis of HSC is difficult, especially when distinguishing it from benign and malignant diseases such as liver abscess, hepatocellular carcinoma and intrahepatic cholangiocarcinoma. Imaging findings and pathological puncture + immunohistochemistry under the guidance of B-ultrasound are helpful for the diagnosis of HSC. Radical surgery is the preferred method for HSC, and postoperative adjuvant chemotherapy is expected to prolong patient survival.

## Author contributions

Conceptualization: Dengyong Zhang, Zheng Lu

Formal analysis: Yang Ma

Investigation: Shuoshuo Ma, Chunshuang Li, Yang Ma

Methodology: Shuoshuo Ma, Chunshuang Li, Yang Ma, Xiaolei Wang

Resources: Shuoshuo Ma, Chunshuang Li, Yang Ma, Xiaolei Wang

Supervision: Dengyong Zhang

Writing–original draft: Shuoshuo Ma, Chunshuang Li

Writing—review & editing: Dengyong Zhang, Zheng Lu

## Acknowledgment

Thanks to each author of the article for their hard work.

## Footnotes

*Patient still alive at last follow-up.
